# Making Our Preference Known: Preference Signaling in the Emergency Medicine Residency Application

**DOI:** 10.5811/westjem.2021.10.53996

**Published:** 2021-12-17

**Authors:** Alexis E. Pelletier-Bui, Benjamin H. Schnapp, Liza G. Smith, Doug Franzen, Elizabeth Barrall Werley, Erin McDonough, Melanie Camejo

**Affiliations:** * Cooper Medical School of Rowan University, Department of Emergency Medicine, Camden, New Jersey; † University of Wisconsin School of Medicine and Public Health, Department of Emergency Medicine, Madison, Wisconsin; ‡ University of Massachusetts Medical School – Baystate, Department of Emergency Medicine, Springfield, Massachusetts; § University of Washington Medical School, Department of Emergency Medicine, Seattle, Washington; ¶ Penn State Health Milton S. Hershey Medical Center, Department of Emergency Medicine, Hershey, Pennsylvania; || University of Cincinnati College of Medicine, Department of Emergency Medicine, Cincinnati, Ohio; # University of Missouri – Kansas City/Truman Medical Center, Department of Emergency Medicine, Kansas City, Missouri

The number of applications to individual emergency medicine (EM) residency programs has markedly increased over the past decade.^1^–^3^ As a result, residency programs have difficulty reviewing applications holistically and struggle to identify applicants who are truly interested in their program. These challenges were exacerbated by the COVID-19 pandemic: programs received more applications; and away-rotation restrictions limited EM applicants’ ability to express, and programs to identify, interest in a residency program or geographic region.^2^ Additionally, the Association of American Medical Colleges reported a concern for maldistribution of interview offers to the highest tier applicants, leaving other well-qualified students with a paucity of interviews – a trend that would threaten the success of the Match for all stakeholders.^4^

The graduate medical education community has made several proposals and implemented innovations in the residency application process in an attempt to help programs identify best fit applicants with the highest likelihood of matching into their program. Some of these innovations, such as the Standardized Video Interview in EM and the required secondary application essay in otolaryngology (ENT), proved ineffective.^5^,^6^ Other practices, such as application filter use, increasing costs per application, implementing caps on applications or interviews, and early or phased cycle matches potentially exacerbate existing inequities for applicants, particularly those who are under-represented in medicine, financially disadvantaged, or lacking mentorship.^7^–^9^ One new innovation, preference signaling (PS), has the potential to be fair and equitable for all applicants as well as low cost and low effort for both residency programs and applicants alike.

Preference signaling is a concept rooted in game theory and developed in labor economics to address the challenge of employers not being able to perform a detailed analysis of all potential applicants and aiding them with identifying high-yield employee prospects. Preference signaling allows applicants to assign virtual “tokens” to their most desired employers, providing applicants the opportunity to communicate their interest, and employers the ability to focus their attention on these most “serious” applicants.^10^

While used by the American Economic Association since 2006, PS only recently gained attention in the residency application process. It was first proposed in the orthopedics and ENT literature in 2017 and 2018, respectively, followed by a promising computer simulation model by ENT in 2019, and implementation by ENT during the 2020–2021 application season.^11^–^14^ During the 2020–2021 application season, each applicant to ENT was able to assign five allotted tokens to desired programs via the Otolaryngology Program Directors Organization (OPDO) website over a two-week period, after which their list was finalized. The OPDO then distributed the lists to individual programs on the same day that the Electronic Residency Application Service (ERAS) opened for application review by programs.^15^ Table 1 shows unpublished preliminary data from the ENT 2020–2021 trial.^14^,^16^ Urology implemented a similar PS program for the 2021–2022 residency application cycle via the American Urologic Association website.^17^ Internal medicine, general surgery, and dermatology have also implemented PS in the 2021–2022 residency application cycle as a component of a supplemental application through ERAS.^18^ Applicants and programs for all participating specialties have the ability to opt out of PS.^15^,^17^,^18^

If managed by a reputable national organization, PS in EM could credibly increase transparency in a process that is high stakes for both applicants and programs, allowing applicants to define program and geographic preference and programs to identify more seriously interested applicants in an equitable manner.^11^,^19^,^20^ It has the potential to provide lower quartile applicants more visibility, when they may otherwise be filtered out of consideration due to low board scores, geography or other factors, and may over time curtail some of the overapplication behavior should applicants discover favorable responses at signaled programs.^16^,^19^ Preference signaling may also attract programs’ attention to applicants previously thought to be “out of their league” and not viable matches.^21^ Allowing the ability to signal preferences with the initial application might also cut down on the amount of time spent on extra applicant communication, such as time spent by applicants drafting emails to specific programs delineating interest, and time spent by program leadership and coordinators responding to those communications. Additionally, it stands to reason that as more applicants receive and accept their most desired interviews (rather than accept offers from less-desired programs), it would potentially relieve interview congestion, opening earlier interviews to middle- and lower- tier applicants.^13^ Similarly, programs could more efficiently assign interview invitations to higher probability matches, potentially reducing interview cancellations.^11^

Some reasonable concerns have been raised about PS. Preference signaling may not actually decrease the number of applications from students.^21^,^22^ By forcing an expression of preference early in the season, PS may disadvantage applicants who may be unclear regarding which programs are a good fit or wish to find their fit in programs during the interview process. Similarly, applicants may change their preferences during the season but would not have the opportunity to reassign their tokens. Programs may hold bias against applicants that do not assign them a token, potentially causing these programs to disregard applicants who may be a strong fit for their environment. Additionally, tokens are valuable due to their scarcity and may be unintentionally devalued by programs that receive a disproportionate number of tokens. It is also worth mentioning that there may be unforeseen challenges or consequences with the PS model for both applicants and programs that have yet to be discovered.

Some important practical considerations must be addressed before PS is implemented. The number of ideal signal tokens per applicant is unclear. The use of too many tokens risks diluting their value and raises the potential for token non-use to be a signal of disinterest. Too few tokens could require applicants to choose arbitrarily between their top programs and may leave programs with too small a pool of signaled applications to make a difference in their review approach. While ENT used five signals during their initial trial, they have decreased this to four for the 2021–2022 application cycle.^14^ Dermatology is using three signals for the 2021–2022 application cycle, whereas internal medicine, surgery, and urology are using five signals.^17^,^18^ Given each of these specialties varies from EM in the number of programs, available positions, number of applicants and average number of applications per applicant, it will be challenging to determine an ideal number of signals based on other specialties’ experience.^1^,^2^,^23^

Of the specialties implementing PS for the 2021–2022 match cycle, general surgery aligns most closely with EM with regard to the number of programs and applicants at 331 and 2908, respectively, compared to 273 and 3734 in EM, but the number of available positions is almost half that in surgery at 1569 compared to 2840 in EM, again making a comparison challenging.^23^. However, given that the use of five tokens is the most common initial start point, we would recommend the same for EM, with adjustments made in future years based on program and applicant feedback as well as Match data.

The best approach for assigning tokens is also unclear. While ENT suggests applicants divide tokens between “reach” programs and programs for which they are competitive, economics research suggests it is ideal to use all tokens on programs where an applicant is competitive.^10^,^24^ Finally, it has been suggested that a continuous-variable system may be more ideal than the current binary PS system, allowing applicants to signal degree of preference in a program by dividing 100 points among prospective programs (eg, all 100 to one program or 20 for each of five programs).^22^

While smaller specialties such as ENT and urology have had success with using their own websites and program director organizations to coordinate PS, we recognize that the much greater number of EM residency programs and applicants to EM may make this exceedingly challenging for the Council of Residency Directors in EM to coordinate and/or finance. Therefore, we would propose using the ERAS platform, as larger specialties like internal medicine and surgery have opted to do. We also support the ENT model of applicants finalizing signals prior to ERAS application opening for programs, which will allow programs to more effectively allocate time to holistic application review and identify high-yield applicants for interview.

Despite these uncertainties, the recurring challenges and current application climate provide a compelling case for trialing PS. We recommend exploring interest in PS from all EM residency application stakeholders, continuing to learn from the experiences of ENT, urology, dermatology, internal medicine, and surgery, and investigating methods for potential implementation of a PS pilot in EM for the 2022–2023 application season. While PS may not decrease the raw number of applications, it could address the largest flaw in the current system: the lack of ability for applicants to communicate, and programs to discern, genuine interest.^19^ By allowing applicants and programs to understand each other better, we believe that PS has the potential to allow for a more sustainable and equitable match process that might create more ideal matches for all candidates and programs with less friction along the way.

**Table 1:** Preliminary preference signaling data from the 2020–2021 otolaryngology application season^a^.

Program data	Applicant data
100% participated (125 programs) 100% received signals ○ The top 10 programs comprised 21% of all tokens ○ Top 20: 38% ○ Top 30: 52% 90% of program directors would continue a similar process in the future (reported after match)	558/632 applicants participated 93% received an interview from ≥ 1 signaled program ○ 15% received interviews from all 5 signaled programs ○ 25% from 4 ○ 21% from 3 ○ 22% from 2 ○ 10% from 1 ○ 7% from 0 Overall, 18% interview offer rate ○ Non-signaled programs: 14% interview offer rate ○ Signaled programs: 58% interview offer rate Lowest quartile of applicants demonstrated 33% increase in interview offers at signaled programs Around 50% of signals are sent to programs from the same geographic region as their home program Fall survey: 70% satisfied/10% dissatisfied 75% would continue a similar process in the future (reported after match)

a Data sources can be found in manuscript references 14 and 16.
